# Sustained delivery of diagnostic and preventive genomics in primary care: a five-year real-world cohort from a family medicine-embedded multidisciplinary clinic

**DOI:** 10.3389/fmed.2026.1850825

**Published:** 2026-06-24

**Authors:** Erika N. Dreikorn, Lucy Galea, Natasha Robin Berman, Christine Munro, Brianne Phillips, Kevin Lee, Lucas A. Berenbrok, Mylynda B. Massart

**Affiliations:** 1 Clinical Translational Science Institute, University of Pittsburgh, Pittsburgh, PA, United States; 2 UPMC Family Medicine, Pittsburgh, PA, United States; 3 Department of Human Genetics, School of Public Health, University of Pittsburgh, Pittsburgh, PA, United States; 4 Department of Pharmacy & Therapeutics, School of Pharmacy, University of Pittsburgh, Pittsburgh, PA, United States; 5 Department of Family Medicine, School of Medicine, University of Pittsburgh, Pittsburgh, PA, United States

**Keywords:** diagnostic genomics, family medicine, genetic counseling, implementation science, multidisciplinary clinic, preventive genomics, primary care genomics, real-world evidence

## Abstract

**Introduction:**

Precision medicine has traditionally been delivered in specialty settings, limiting access for many patients. Primary care offers a potential entry point for broader delivery of genomic services within primary care, given its central role in longitudinal and preventive care. In this study, we characterize five years of sustained real-world delivery of both diagnostic and preventive genomic services within a family-medicine-embedded multidisciplinary clinic, examining patient population, referral patterns, clinical workflow, and genomic testing yield.

**Methods:**

We conducted a retrospective chart review of all new patients evaluated in the PCPM clinic at UPMC between November 6, 2019, and April 17, 2024 (*n* = 1,602). Patients were seen via in-person and telemedicine visits for comprehensive family history assessment, genetic counseling, and clinician evaluation. Data collected included demographics, referral sources and indications, test types, and genetic testing outcomes. Summary statistics were reported using frequencies and proportions.

**Results:**

Genetic testing was ordered for 1,174 patients (73%), with 1,360 total tests ordered and an 83.4% completion rate. Panel-based testing accounted for 71% of all tests. Most tests (86%) were ordered for primary referral concerns, while 14% were prompted by incidental indications identified during intake. Clinic growth supported increased access, expanded staffing, and the development of a streamlined two-visit care model.

**Conclusion:**

These findings demonstrate that the sustained delivery of diagnostic and preventive genomics within a family medicine embedded multidisciplinary clinic model is feasible, enabling broad genomic evaluation, efficient testing workflows, and clinically meaningful diagnoses across diverse indications, offering a replicable template for health systems seeking to close the genomics access gap.

## 1 Introduction

Precision medicine is a healthcare approach that tailors prevention, diagnosis, and treatment strategies to an individual’s unique genetic makeup, environment, and lifestyle, with the goal of improving health outcomes and minimizing harm (1). Precision medicine has gained increasing clinical adoption and is most commonly implemented in specialties such as oncology and prenatal care (2–7). In these settings, care guided by genetics has enabled more accurate diagnoses, targeted therapies, and personalized treatment selection. Importantly, these benefits can extend beyond specialty care alone (8–10). Despite this broad potential for impact across healthcare, widespread implementation remains limited due to challenges such as high testing costs, disparities in test access, variable insurance coverage, limited provider education, and gaps in integrating genomic data into clinical workflows and electronic health records (11–15). Addressing these barriers is essential to realize the potential of precision medicine and ensure equitable access for diverse patient populations.

Primary care provides a particularly promising setting for advancing the broad application of precision medicine. Because primary care clinicians (PCCs) manage patients’ health across the lifespan and often care for multiple generations within the same family, they are uniquely positioned to integrate individualized genomic insights into routine, longitudinal care (16). This continuity, combined with the coordinating role of PCCs across specialties, enables precision medicine to move beyond condition-specific use and toward a population-level impact. Embedding genetic testing and interpretation within primary care allows for proactive risk assessment, earlier detection of heritable conditions, and cascade testing of at-risk relatives, shifting care from reactive treatment to prevention-focused management. Despite growing consensus that primary care is an appropriate setting for genomics integration, the published evidence base consists predominantly of funded research programs rather than sustained clinical services operating under real-world constraints (15). Evidence that such a model can survive operational realities such as scheduling demand, payer variability, workforce turnover, and workflow evolution over multiple years is largely absent from the literature (10).

The Primary Care Precision Medicine (PCPM) clinic at UPMC represents a real-world example of how precision medicine can be integrated into the primary care setting, as previously described (17). Established in 2019, the clinic evaluates patients for hereditary conditions, provides genetic counseling, coordinates genetic testing for indications such as pharmacogenomics and cancer risk assessment, and delivers results and management recommendations back to patients and their primary care providers. By serving as a centralized resource for genetic expertise while supporting continuity of care within patients’ existing primary care relationships, the PCPM clinic demonstrates a scalable model for achieving current guideline-driven genetic testing such as hereditary cancer, and carrier screening to embedding emerging precision medicine such as multi-cancer early detection into routine practice.

Over the past five years, the PCPM clinic has steadily evolved to meet a growing demand for precision medicine services in the primary care setting. What began as a limited, in-person service with a small multidisciplinary team has expanded into a full-time clinic supported by multiple physicians, certified genetic counselors, a nurse practitioner, and a pharmacogenomics-trained pharmacist. Alongside this growth, the clinic has streamlined its approach to patient care, enabling broader patient access while maintaining comprehensive genetic evaluation and counseling. This evolution reflects the rising demand for precision medicine in primary care and demonstrates the feasibility of expanding this clinic model within a large health system. Importantly, the success of the PCPM clinic has been closely tied to the specialized expertise of its multidisciplinary team. Team members were equipped, through formal training and clinic-based learning, to develop and apply the clinical, genetic counseling, and pharmacogenomics skills necessary to support this integrated care model. While such expertise is not universally available in all primary care settings, these findings highlight the importance of targeted workforce training, continuing education, and embedded genetics support as key strategies to enable broader replication of this approach. Importantly, the growth of the clinic reflected not only the fulfillment of its original mission to evaluate diagnostic and preventive genomic testing within primary care, but also two unanticipated evolutions in clinical scope that emerged organically in response to demonstrated health system need. First, as the options for genetic explanation of disease in adult medicine have expanded, non-genetics providers across adult specialties have increasingly required support for genetic testing selection, ordering coordination, informed patient consent and result interpretation, functions that fall outside the typical training and bandwidth of most clinicians. The PCPM clinic came to fulfill this facilitative role, functioning as a consultative genomics resource for the health system’s adult medicine providers who are seeking diagnostic genetic evaluation but do not have the infrastructure to independently manage all aspects of that process. Secondly, a substantial and unanticipated demand for connective tissue disorder (CTD) evaluation, encompassing both hypermobility-spectrum presentations and vascular CTDs, which emerged and grew organically from patient and provider need rather than deliberate programmatic planning, and ultimately represents a significant portion of the clinic’s volume.

To address the limited real-world evidence on how precision medicine can be effectively implemented in primary care, we conducted a retrospective chart review of all new patients seen at the PCPM Clinic between November 2019 and April 2024. This analysis provides real-world operational evidence for the feasibility and sustainability of a family-medicine–housed, multidisciplinary genomics service delivering both diagnostic and preventive genomic care, over five years of routine clinical operations. We describe the patient population, referral patterns, workflow evolution, and genetic testing yield to characterize this model as a replicable template for health systems working to close the genomics access gap. In doing so, it highlights both the opportunities and challenges of integrating precision medicine into routine patient care and offers insights to guide future efforts aimed at scaling and sustaining such models.

## 2 Materials and methods

### 2.1 Study population

All new patients (*n* = 1,602) seen at the PCPM Clinic between November 6, 2019, and April 17, 2024, were included in this retrospective chart review, encompassing both in-person and telemedicine appointments. All medical records were evaluated by authors E.D. or L.G. to document overall metrics for the clinic since its inception. Summary statistics were calculated and reported as frequencies and proportions.

### 2.2 Structure and sustained evolution of a family-medicine-embedded genomics clinic

The PCPM clinic at UPMC was conceptualized in 2019 by M.M., P.E., and J.S. The clinic initially began by operating as an in-person service for two hours per week. The founding team consisted of one primary care physician (M.M.), who is also trained in molecular biology, two genetic counselors (C.M. and N.R.B.), a pharmacogenomics (PGx)-trained pharmacist (L.B.), and was led by an administrative manager (J.S.). During this early phase, patients typically had three visits related to genetic testing. In the first visit, they met with both the physician and a genetic counselor to review a comprehensive family history and explore their primary reason for referral as well as potential areas for further genetic evaluation beyond their initial referral reason. The second visit involved consenting for any genetic testing recommended by the team, and the third visit was dedicated to reviewing the genetic test results. At this final appointment, the genetic counselor explained the results, while the physician discussed their clinical significance and co-developed a management plan based on the findings. The clinic expanded to one half day after the first four months and then pivoted to telemedicine in March of 2020 with the onset of the pandemic.

The clinic has not engaged in formal marketing or advertising; awareness has grown through organic word of mouth, with UPMC providers learning of the clinic through grand rounds, departmental communications, and colleague recommendations, and patients learning of the clinic through prior PCPM patients, provider recommendations, and online patient communities focused on connective tissue disorders, dysautonomia, and complex chronic illness. The clinic accepts both UPMC-affiliated and non-UPMC patients, with self-pay and out-of-network arrangements accommodated where possible. The substantial self-referral volume also reflects a structural feature of embedding genomic care within primary care: unlike specialty or genetics services, which typically require a formal physician referral, most insurance plans permit direct self-referral to primary care. This lowers the access threshold for patients who lack a referring provider, a particularly consequential barrier for those with complex or underrecognized presentations such as hypermobility spectrum disorders. A substantial share of self-referrals and rheumatology referrals were patients seeking evaluation for CTD symptoms, many of whom had been unable to access evaluation elsewhere because CTD assessments, particularly for hypermobile Ehlers-Danlos syndrome (hEDS), are frequently declined by specialty genetics clinics due to perceived limited diagnostic utility of available testing.

Over time, the PCPM clinic has expanded its team in response to patient scheduling demand, to now include two physicians (M.M. and K.L.), a nurse practitioner (B.P.), three genetic counselors (C.M., N.R.B., and L.G.), a PGx-trained pharmacist (L.B.), two administrative staff (J.S. and M.K.), and a research director (E.D.). In addition, the clinic routinely hosts medical students, genetic counseling students, and pharmacogenomics fellows, who participate in patient care under direct supervision, contributing to both service delivery and workforce development in precision medicine. With this growth, the clinic now operates five days a week and has streamlined its patient process into a two-visit model. During the first visit, patients meet with a genetic counselor to review their family history and discuss the primary referral reason, as well as any additional areas for testing based on risk factors identified in the family history. The patient also meets with a clinician, who performs any relevant physical examination and confirms personal history. If genetic testing is indicated, the genetic counselor will obtain consent for testing during this same visit. The second visit occurs once the genetic test results are available, during which the patient meets with the genetic counselor and either the physician or nurse practitioner to review the clinical significance of the results, discuss management plans, and arrange referrals to specialist clinics as necessary. The PCPM clinicians (MD, DO, NP) function as precision medicine specialists embedded within a primary care framework: they directly evaluate patients, perform relevant physical examinations, integrate genetic findings into the broader clinical context, and communicate findings and recommendations back to the patient’s own primary care clinician or referring provider. The PCPM team serves as a consultative resource rather than an ongoing care provider; longitudinal care is directed by the patient’s referring clinician or primary care provider, who retains responsibility for long-term management, including ordering of surveillance and specialist follow-up. The PCPM team remains available for follow-up questions and guidance as genetic findings evolve, but does not assume ongoing clinical liability beyond the scope of the genomic evaluation and its directly communicated recommendations. When findings warrant specialist referral, such as for confirmed hereditary cancer syndromes requiring subspecialty surveillance or connective tissue disorders with multisystem involvement, this recommendation is communicated to the referring or primary care provider, who coordinates the referral as appropriate.

### 2.3 Diagnostic and preventive genomic testing protocols

After the genetic test order is placed, the testing company will typically send a buccal sample collection kit directly to the patient or will coordinate with a third-party company to collect a mobile phlebotomy sample. All clinical testing ordered by the PCPM clinic is completed in a Clinical Laboratory Improvement Amendments (CLIA) and Certified Analytics Professional ® (CAP) – certified laboratory, which included Ambry Genetics (Aliso Viejo, California), Athena Diagnostics (Marlborough, Massachusetts), Blueprint Genetics (Seattle, Washington), Color Health (Burlingame, California), Fulgent Genetics (Temple City, California), Galleri (Menlo Park, California), GeneDx (Gaithersburg, Maryland), GeneSight (Mason, Ohio), Invitae (San Francisco, California), Labcorp (Burlington, North Carolina), MNG Laboratories (Atlanta, Georgia), Myriad Genetics (Salt Lake City, Utah), Natera (Austin, Texas), OneOme (Minneapolis, Minnesota), PerkinElmer (Waltham, Massachusetts), Prevention Genetics (Marshfield, Wisconsin), Quest (Secaucus, New Jersey), Sema4 (Stamford, Connecticut), UAB Medicine (Birmingham, Alabama), UPMC Genome Center (Pittsburgh, Pennsylvania), UPMC Magee Lab (Pittsburgh, Pennsylvania), and Variantyx Inc. (Framingham, Massachusetts). Genetic testing was chosen for its appropriateness based on the patient’s indication, gene content of the panel, technology inclusions (e.g., RNA sequencing or repeat expansion), billing considerations such as cost estimates provided prior to testing and financial assistance programs, and ease of sample collection (buccal swab or mobile phlebotomy options). Consistent genetic testing workflows between all clinicians, and the continual reassessing of testing being offered, ensured equitable access to genetic testing for patients, regardless of income or socioeconomic status. Genetic testing methodologies ordered included karyotype, chromosomal microarray, single-gene testing, multigene panels, repeat expansion analysis, exome sequencing (ES), and genome sequencing (GS). Variant interpretation reports were provided by the companies who performed the genetic testing; interpretation and clinical application were discussed at weekly patient review meetings by the clinic team. For patients with testing ordered for indications outside of cancer, CTD, and familial hypercholesterolemia, final clinical correlation and management was left to the referring specialist. Testing results ranged across the continuum of possible outcomes, including negative results, benign or likely benign findings, carrier status, pathogenic or likely pathogenic variants, and variants of uncertain significance (VUS).

All patients were consented for genetic testing by a genetic counselor or clinician. Informed consent included discussion about the basics of genes and DNA, what the testing is looking for (e.g., single gene, panel, exome, or genome), condition related information, inheritance pattern/s, types of results, GINA, and the logistics of sample collection and billing. If the patient was being consented for exome or genome sequencing, consent also included, as offered by the testing laboratory, for opt in/out options including ACMG secondary findings, incidental findings, PGx results and/or research opportunities. Consent was given by patients for each individual test ordered, and was re-obtained in the instance of re-requisition of panel testing or reanalysis of exome or genome sequencing. As most patients are seen by telemedicine, verbal consent is documented in the clinician and genetic counselor notes.

## 3 Results

### 3.1 Clinic volume and patient demographics

**Figure 1:**
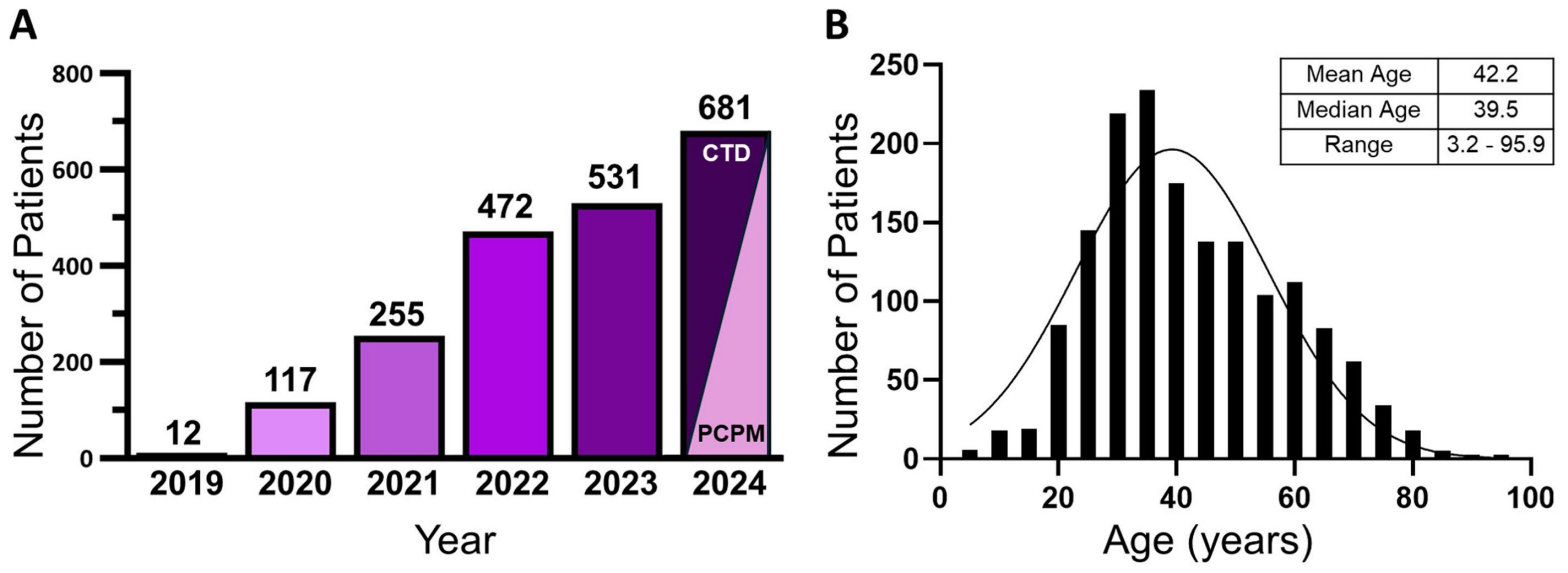
Growth and age demographics of the PCPM clinic. **(A)** Annual number of new patients seen at the PCPM clinic from 2019 to 2024. In 2024, the clinic divided appointment slots between patients referred for connective tissue disorder (CTD) evaluations and traditional PCPM referrals due to increased CTD patient volume. **(B)** Frequency distribution of patient age at their first visit to the PCPM clinic, with a Gaussian (normal) curve fit. Age is shown in years.

Between November 2019 and April 2024, the PCPM Clinic at UPMC evaluated 1,602 patients through both telehealth and in-person appointments, with patient volume increasing steadily over time (Figure 1A). The average age at first visit was 42.2 years (median: 39.5 years), with patients ranging in age from 3.2 to 95.9 years (Figure 1B). Demographic information, such as race/ethnicity, education, socioeconomic status, and geographic distribution, were not collected during this chart review.

A summary of patient gender and insurance type is provided in Table 1. The majority of patients identified as female (71.4%), followed by male (24.8%), nonbinary (2.5%), transgender male (0.8%), and transgender female (0.4%). Insurance coverage varied, with most patients insured through commercial plans, followed by Medicaid, Medicare, and other payer types.

**Table 1:** Distribution of gender and insurance type among PCPM patients.

Patients	Number	(%)
Gender		
Male	398	24.84%
Female	1,144	71.41%
Nonbinary	40	2.50%
Transgender male	13	0.81%
Transgender female	7	0.44%
Insurance type		
Commercial	820	51.19%
Medicaid	280	17.48%
Medicare	145	9.05%
Multiple	238	14.86%
Uninsured	106	6.62%
Military	13	0.81%

### 3.2 Referring providers and referral reasons

**Figure 2:**
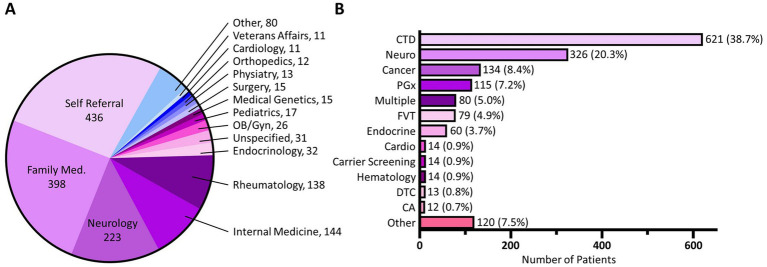
Referral sources and referral indications for PCPM patients. **(A)** Distribution of patient referrals to the PCPM clinic, with the majority originating from self-referrals, family medicine, and neurology. The “Other” category includes referrals from a range of specialties, such as gastroenterology (GI), naturopathy, physical therapy, psychiatry, hematology, dermatology, oncology, nephrology, social work, geriatric medicine, plastic surgery, immunology, emergency medicine, prenatal group, pulmonology, radiology, and urology. **(B)** Distribution of referral reasons, with the highest proportion of patients referred for connective tissue disorder (CTD) concerns (621, 38.7%) and neurology concerns (326, 20.3%). PGx = Pharmacogenomics; FVT = familial variant testing; DTC = referral regarding direct-to-consumer testing; CA = chromosomal abnormality.

Patients being seen by the PCPM clinic come from a variety of specialties, as well as a large number of self-referral patients. Figure 2A shows the distribution of referring providers, with the majority being self-referrals (436 patients, 27.2%) or referrals from family medicine (398 patients, 24.8%), neurology (223 patients, 13.92%), internal medicine (144 patients, 9.0%), and rheumatology (138 patients, 8.6%). Other specialty referrals included endocrinology, OB/Gyn, pediatrics, medical genetics, surgery, physiatry, orthopedics, cardiology, Veterans Affairs, gastrointestinal, naturopath, physical therapy, psychiatry, hematology, dermatology, oncology, nephrology, social work, geriatric medicine, plastic surgery, immunology, emergency medicine, prenatal genetics, pulmonology, radiology, and urology. Figure 2B illustrates the distribution of referral reasons among patients, with the majority being referred for concerns related to CTD. CTD evaluations in this clinic encompass heritable connective tissue disorders including hEDS and related conditions; CTD-specific genetic testing here is used to rule out a monogenic cause (e.g., Marfan, Loeys-Dietz, classical EDS) rather than to confirm a clinical diagnosis. The “Other” category includes referrals for renal, rheumatologic, dermatologic, GI, immunologic, pulmonary, and metabolic conditions, along with various other concerns.

### 3.3 Evaluation, genetic testing, and diagnostic yield

**Figure 3:**
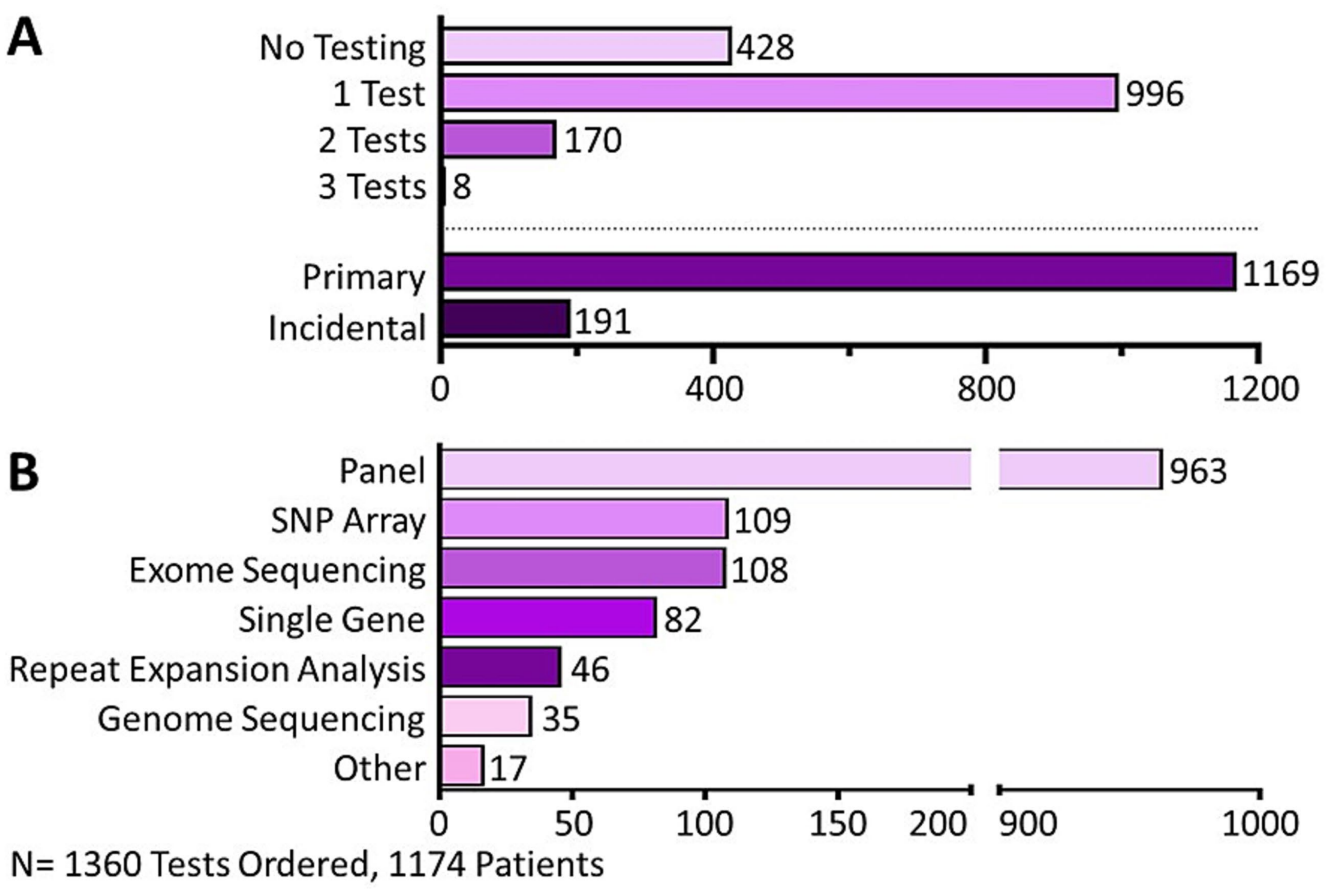
Genetic tests ordered at PCPM clinic. **(A)** Number of tests ordered per patient, categorized by whether testing addressed the primary referral reason or an incidental indication identified during evaluation by the genetic counseling team. **(B)** Distribution of the types of genetic tests ordered during patient encounters (*N* = 1,360 tests among 1,174 patients).

Of the 1,602 new patients seen at the PCPM clinic, genetic testing was ordered for 1,174 individuals (73%), while 428 patients (27%) did not meet criteria for testing. Patients were considered not to meet criteria for genetic testing when clinical evaluation did not identify sufficient personal or family history to support testing, when available testing was unlikely to provide clinically actionable information based on the presentation, or when testing had already been completed elsewhere and the visit was primarily intended for genetic counseling and result interpretation. These patients still received a full evaluation including pedigree assessment, genetic counseling, and clinician review, and were provided with guidance on family history-based risk management, recommendations for clinical surveillance where appropriate, and information about when initial or additional testing might become warranted. In total, 1,360 genetic tests were ordered. Most individuals (996) had a single test, while 170 had two tests, and 8 individuals had three tests (Figure 3A). Notably, some tests were ordered in response to heritable risk factors identified during the genetic counselor’s comprehensive pedigree assessment, representing the preventive genomic function of the clinic, in which proactive family-history evaluation surfaces actionable risk beyond the primary referral complaint.

Of the 1,360 tests, 1,169 (86%) were ordered for the patient’s primary reason for referral, whereas 191 tests (14%) were ordered based on additional heritable risk factors identified during intake (Figure 3A). As shown in Figure 3B, the majority of tests ordered were panel-based (963 tests, 71%), reflecting the clinic’s emphasis on broad, clinically actionable assessments. “Other” tests include Multicancer Early Detection (Galleri), Karyotype, and Chromosomal Microarray (CMA). Among the tests ordered, the cohort included a meaningful volume of tests targeting CDC Tier 1 genomic conditions, which represent the highest-evidence actionable findings in clinical genomics: 37 tests were ordered for hereditary cancer syndromes (BRCA1/2-related hereditary breast and ovarian cancer and Lynch syndrome), 5 for familial hypercholesterolemia, 125 for pharmacogenomics, and 153 for population-level screening panels encompassing cardiovascular risk, hereditary cancer, and pharmacogenomics, together comprising 320 tests (23.5% of all tests ordered).

### 3.4 Testing summary and genetic testing results

Of the 1,360 tests ordered by the PCPM clinic, 1,134 (83.4%) were completed, while 226 (16.6%) were not. To examine the diagnostic yield of all testing ordered, it was further divided into general (non-CTD) testing and CTD-specific testing. There were 851 tests ordered for general concerns not related to CTD, 698 (82.0%) of which were completed, while 153 (18.0%) were not completed. 509 CTD-specific tests were ordered, with 436 (85.7%) completed and 73 (14.3%) not completed.

**Figure 4:**
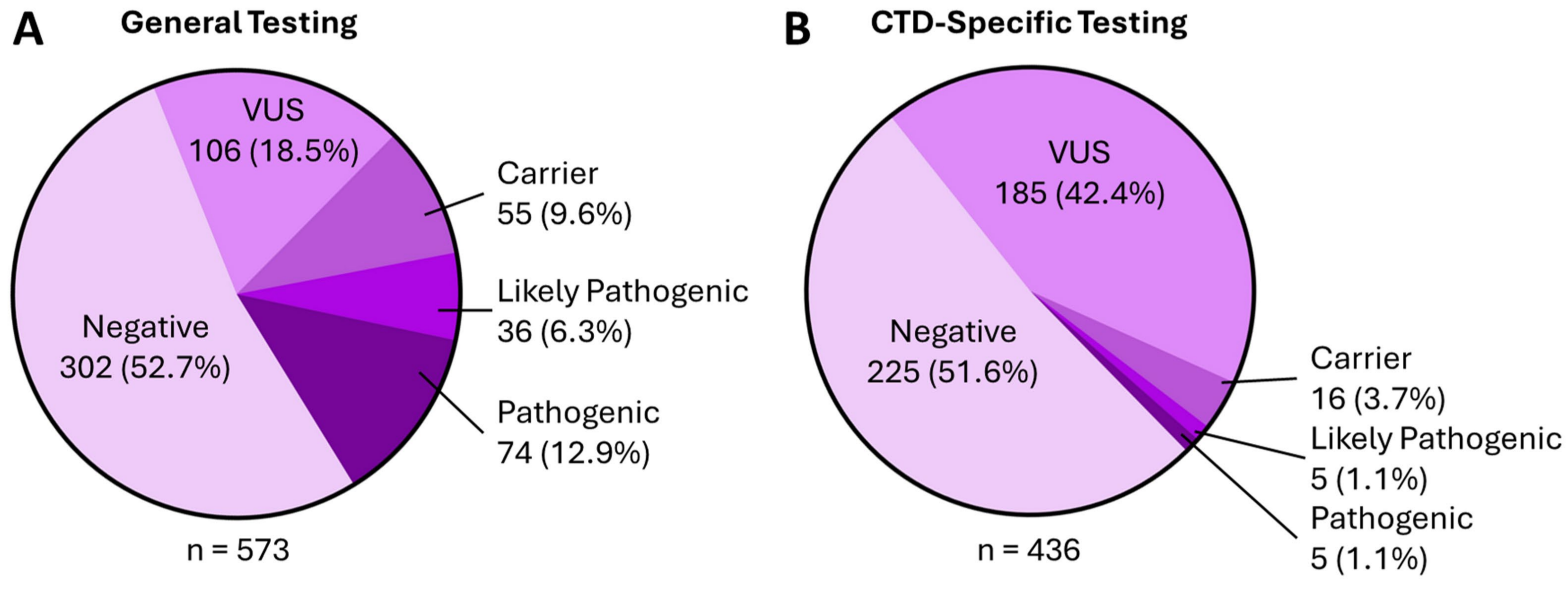
Genetic testing completion and results summary. **(A)** Results of general genetic testing (*n* = 573), excluding PGx testing. Results are categorized as Negative, Variants of Uncertain Significance (VUS), Carrier, and Likely Pathogenic (LP) or Pathogenic. **(B)** Results of CTD-specific genetic testing (*n* = 436). Result categories mirror those in panel **A**.

Pharmacogenomic (PGx) results (125 tests) were excluded from this analysis, as PGx reports reflect metabolizer status classifications and gene-drug interaction profiles rather than standard variant pathogenicity categories, and are therefore not directly comparable to diagnostic genetic testing outcomes. After excluding these results, 573 general (non-CTD) genetic test results remained. Among these, 302 results (52.7%) were negative, 106 (18.5%) were classified as variants of uncertain significance (VUS), 36 (6.3%) were likely pathogenic, 74 (12.9%) were pathogenic, and 55 (9.6%) identified carrier status (Figure 4A). For CTD-specific genetic testing, 436 results were available. Of these, 225 (51.6%) were negative, 185 (42.4%) were classified as VUS, 16 (3.7%) indicated carrier status, and 5 each (1.2%) were classified as pathogenic and likely pathogenic, respectively (Figure 4B). For both testing groups, these results represent the first and most significant result. For example, if an individual had a pathogenic result and a VUS, only the pathogenic result is considered here.

## 4 Discussion

To our knowledge, the PCPM clinic represents one of the few published examples of a sustained, real-world genomic medicine service operating continuously over a multi-year period without dedicated research infrastructure. Unlike the large genomics research cohorts that dominate the implementation literature, this service has operated under routine clinical constraints, including evolving payer mix, staffing growth driven by demand rather than grant cycles, and workflow adaptation in response to operational pressures including a pandemic. The five-year sustainability and growth of this model is itself an implementation science finding, demonstrating that a genomics service of this scope can survive the realities of clinical operations. The breadth of referral indications from connective tissue disorders and neurological concerns to cardiology, oncology, endocrine, and hematologic issues, highlights how patients naturally bring a wide range of genetic questions to their primary care providers. Because primary care clinicians routinely encounter diverse symptoms across the lifespan and often care for multiple generations of the same family, they are well-positioned to identify individuals who may benefit from genetic evaluation. Our findings support that primary care can serve as an effective entry point for precision medicine when supported by an integrated and skilled team.

A central strength of the PCPM clinic is its multidisciplinary structure, which enables a broader scope of care beyond what would typically be expected in a primary care setting. Genetic counselors provide detailed pedigree assessments, ensure informed consent, and guide test selection, clinicians integrate genetic findings with patients’ broader medical needs, and a pharmacogenomics-trained pharmacist supports medication management decisions. This collaborative expertise allows the clinic to address diverse referral reasons while ensuring appropriate testing, interpretation, and clinical follow-up. Importantly, the integrated team model facilitates identifying additional testing needs that may not be apparent at the time of referral. The fact that 14% of tests were ordered due to incidental findings uncovered during comprehensive family history evaluation underscores how multidisciplinary assessment adds meaningful diagnostic depth that may be difficult to achieve in standard referral-based pathways.

The observed testing patterns in this cohort further illustrate the breadth of precision medicine applications within this primary care model. Most patients underwent a single genetic test; however, 178 individuals had multiple tests ordered to evaluate incidental indications or to pursue further assessment when initial testing did not yield a diagnostic result. Multigene panel testing was the most commonly ordered genetic test, reflecting both contemporary best practices and the practical need to address diverse differential diagnoses efficiently. Diagnostic yield varied by testing category, with pathogenic or likely pathogenic variants identified across both general and CTD-specific testing groups. As anticipated, CTD-focused testing yielded a substantial proportion of variants of uncertain significance, consistent with current limitations in variant interpretation in connective tissue disorders.

The predominance of CTD referrals in this cohort warrants specific comment. CTD referrals were driven largely by two factors: a high volume of self-referrals from patients with suspected heritable connective tissue disorders who had encountered difficulty accessing evaluation elsewhere, and a significant number of referrals from specialties, such as rheumatology, seeking genetic evaluation to rule out a monogenic etiology in patients presenting with CTD symptoms. Many specialty genetics services decline to evaluate patients with hypermobility-related presentations due to the limited diagnostic yield of available genetic testing in this group. The PCPM clinic’s willingness to engage this population, providing thorough genetic counseling, appropriate targeted testing, and clear communication of results and their limitations, has positioned it as a recognized resource for a patient group with substantial unmet needs. Examining CTD referrals by sub-type reveals distinct patterns that reflect the breadth of the unmet need this clinic has come to address. Vascular CTD referrals were predominantly referrals initiated by cardiovascular surgery, vascular surgery, and cardiology specialists following an index vascular event, such as aortic dissection, aneurysm, spontaneous arterial dissection, or identification of concerning imaging findings. These referrals reflect a growing recognition among adult vascular specialists that heritable connective tissue disorders warrant genetic evaluation for risk stratification, cascade family testing, and surgical management planning, yet lack a clear clinical home within existing genetics infrastructure.

Hypermobility-related evaluations, by contrast, were referred through a diverse network of providers including rheumatology, orthopedics, physical therapy, and primary care, as well as through a substantial volume of self-referrals from patients who had navigated to the PCPM clinic through peer communities and social media networks dedicated to complex chronic illness. The convergence of these two referral streams illustrates how the clinic evolved in response to demonstrated and unmet patient need across adult medicine rather than through deliberate program design. Beyond demonstrating unmet need, the volume and clinical success of CTD referrals, particularly hypermobility-related evaluations, support a broader argument about where this care should be situated. Patients with suspected heritable connective tissue disorders, and hypermobility spectrum presentations in particular, are frequently declined or deprioritized by specialty genetics services because of limited diagnostic yield, yet they require precisely the elements that primary care is built to deliver: longitudinal relationships, multisystem assessment, coordination across rheumatology, orthopedics, cardiology, and physical therapy, and management of chronic symptoms in the absence of a unifying molecular diagnosis. The PCPM clinic’s experience suggests that hypermobility and related CTD presentations can be appropriately triaged and, in many cases, diagnosed within a primary care setting when supported by integrated genetic counseling expertise, pedigree-based risk assessment, application of established clinical criteria (e.g., 2017 hEDS criteria, Ghent and Beighton frameworks), targeted molecular testing to rule out monogenic causes, and clear communication about the limits of current testing. Framing these conditions as primary-care–appropriate, rather than as residual cases left over when specialty services decline, reorients the clinical pathway around the workflow that already serves these patients best.

As patient demand increased, the PCPM clinic adapted its care model to maintain accessibility without sacrificing clinical quality. The clinic transitioned from a three-visit structure to a streamlined two-visit process that preserved comprehensive evaluation while improving efficiency. Expanding clinic availability from limited weekly hours to full-time operations, alongside growth in clinical staffing driven by patient demand, further increased access to genetic services. These workflow modifications demonstrate how operational refinement can support scalability within a large health system while maintaining multidisciplinary collaboration. The clinic’s flexibility was further demonstrated by its successful shift to telemedicine during the COVID-19 pandemic, reinforcing the role of virtual care as a valuable strategy for expanding the reach of precision medicine within primary care settings.

A key component driving the clinic’s diagnostic success is the use of detailed pedigree assessment as a routine element of evaluation. A broad family history collection allowed genetic counselors and clinicians to detect risk patterns not captured by the original referral indication. The frequency of incidental testing orders underscores the limitations of focused, referral driven pedigrees, and emphasizes the central role of comprehensive pedigree analysis in identifying heritable risk across disease categories. In this model, the pedigree became not merely a documentation tool, but a diagnostic instrument that expanded clinical insight and facilitated proactive testing and cascade screening of at-risk relatives. This unified approach, in which a single pedigree-driven evaluation surfaces both the primary referral concern and additional heritable risk, distinguishes PCPM from most genomics services, which are designed for either diagnostic or preventive indications but rarely both within a single workflow. The 14% incidental indication testing rate represents patients who would have required a referral to a separate service in most health systems, and in many cases would never have received that second referral at all.

The PCPM model also offers a partial answer to the genomics workforce shortage that has been identified as a major barrier to broader implementation. The genetics workforce cannot scale fast enough to meet growing demand for genomic services, and calls for alternative service delivery models have been persistent in the literature (18, 19). Rather than relying on expansion of specialty genetics infrastructure, PCPM embeds certified genetic counselors and a pharmacogenomics-trained pharmacist within a family medicine department, led by clinicians/providers with primary care continuity-of-care training. This staffing model is designed for replication: it does not require a tertiary academic medical center, a dedicated genetics department, or research funding, only a committed multidisciplinary team and institutional support for embedding genetics expertise within primary care workflows. The payer diversity of this cohort, including 17% Medicaid, 7% uninsured, and 15% multiple-payer patients, suggests that this model can serve populations who are typically underrepresented in published genomics cohorts, a finding consistent with the clinic’s deliberate approach to cost-conscious test selection and engagement with financial assistance programs (13).

This study has several limitations that should be considered when interpreting these findings. First, as a single-center retrospective chart review conducted within one large academic health system, the generalizability of these findings to other practice settings, particularly community-based, rural, or resource-limited primary care environments, may be restricted. Second, the high proportion of self-referrals (27%) suggests that patients accessing the PCPM clinic may disproportionately represent health-seeking individuals with greater health literacy, awareness of genetic services, and motivation to pursue testing. This self-selection bias, combined with limited outreach pipelines to referring providers outside the UPMC system, may limit the extent to which the clinic’s patient population reflects the broader primary care population who could benefit from precision medicine services. Third, demographic information including race/ethnicity, education level, socioeconomic status, and geographic distribution were not systematically collected as part of this chart review, precluding analysis of health equity dimensions and limiting our ability to assess whether the clinic is reaching diverse and underserved communities. Fourth, as this was a retrospective chart review, all data, including insurance type and referring provider, reflect information recorded in the electronic health record at the time of data collection rather than at the time of each clinical encounter. As a result, some data points may reflect changes that occurred between the patient’s visit and the chart review, and incomplete EHR documentation in a subset of records, most notably the absence of a listed referring provider in some cases, may introduce missing data that could affect the accuracy of referral source reporting. Fifth, as with all multigene panel testing, a substantial proportion of results, particularly within CTD-specific testing, were classified as variants of uncertain significance (VUS). While VUS results are managed through ongoing clinical surveillance and reviewed at weekly team meetings, their classification may change over time as the evidence base for variant interpretation evolves, and their clinical utility at the time of return is limited; patients are counseled accordingly and advised to follow up periodically as reclassification data become available. Finally, this analysis captures clinic metrics and testing outcomes over a five-year period but does not include long-term follow-up data on downstream clinical outcomes, such as the uptake of recommended surveillance, the impact of results on patient management, or health outcomes among at-risk relatives identified through cascade testing. Future prospective studies with standardized demographic data collection and longitudinal outcome tracking will be essential to fully evaluate the impact of this care model. This real-world cohort demonstrates that the sustained delivery of both diagnostic and preventive genomics is achievable within a family-medicine-embedded multidisciplinary clinic structure, serving a payer-diverse population across five years of uninterrupted clinical operations. The staffing model, unified pedigree-driven workflow, and operational benchmarks reported here constitute an evidence-grounded template for health systems seeking to expand genomics access in the absence of near-term specialty genetics workforce growth.

## Data Availability

The original contributions presented in the study are included in the article/supplementary material, further inquiries can be directed to the corresponding author.
